# Comparing the Currents Measured by CARTHE, CODE and SVP Drifters as a Function of Wind and Wave Conditions in the Southwestern Mediterranean Sea

**DOI:** 10.3390/s22010353

**Published:** 2022-01-04

**Authors:** Pierre-Marie Poulain, Luca Centurioni, Tamay Özgökmen

**Affiliations:** 1 NATO Science and Technology Organization, Centre for Maritime Research and Experimentation, 19126 La Spezia, Italy; 2 Scripps Institution of Oceanography, University of California San Diego, La Jolla, CA 92093, USA; lcenturioni@ucsd.edu; 3 Rosenstiel School of Marine and Atmospheric Science, University of Miami, Miami, FL 33149, USA; tozgokmen@rsmas.miami.edu

**Keywords:** near-surface ocean currents, drifters, Acoustic Doppler Current Profiler

## Abstract

Instruments drifting at the ocean surface are quasi-Lagrangian, that is, they do not follow exactly the near-surface ocean currents. The currents measured by three commonly-used drifters (CARTHE, CODE and SVP) are compared in a wide range of sea state conditions (winds up to 17 m/s and significant wave height up to 3 m). Nearly collocated and simultaneous drifter measurements in the southwestern Mediterranean reveal that the CARTHE and CODE drifters measure the currents in the first meter below the surface in approximately the same way. When compared to SVP drogued at 15 m nominal depth, the CODE and CARTHE currents are essentially downwind (and down-wave), with a typical speed of 0.5–1% of the wind speed. However, there is a large scatter in velocity differences between CODE/CARTHE and SVP for all wind and sea state conditions encountered, principally due to vertical and horizontal shears not related to the wind. For the CODE drifter with wind speed larger than 10 m/s and significant wave height larger than 1 m, about 30–40% of this difference can be explained by Stokes drift.

## 1. Introduction

Since the advent of satellite positioning and data telemetry in the late 1970s, instrumented buoys freely-drifting at the ocean surface have become increasingly popular to measure meteo-marine properties near the air–sea interface. In particular, they have been designed with a drogue to measure currents near the ocean surface, with varying levels of accuracy, by tracking their positions in time in order to investigate upper ocean dynamics over a wide range of spatial scales, from the global ocean [1,2] to marginal seas [3,4,5] and small coastal areas [6,7], and at time scales of hours to decades.

However, drogued drifting buoys, hereafter referred to as drifters, do not exactly follow the water around them, and the estimation of horizontal velocities from their displacements is prone to systematic errors. This is especially true in high sea conditions, when winds, surface waves and vertical shear in the upper water column can produce significant slippage of the drifters. In addition, being constrained at the sea surface, drifters are obviously unable to measure vertical velocities. Vertical velocities can, however, be inferred from surface divergence using the continuity equation (e.g., [8]).

Generally, the cross-section of the surface expression of a drifter above the sea surface and exposed to winds is minimized with respect to the section of the underwater tether and drogue to reduce the direct wind drag. Studies have also been conducted on the mechanical characteristics of the tether and on the shape, size and depth of the drogue in order to limit the impact of slippage and wave rectification on the drifter (e.g., [9,10]).

In this paper, we compare the ocean currents measured by three standard drifters: the Consortium for Advanced Research on Transport of Hydrocarbon in the Environment (CARTHE), the Coastal Ocean Dynamics Experiment (CODE) and the Surface Velocity Program (SVP) designs, using data collected in the southwestern Mediterranean with winds as large as 17 m/s and waves with significant wave height up to 3 m. Since the near-surface shear associated with the balanced geostrophic flow is usually small, differences of currents measured by the drifters at different depths in the upper ocean are mainly be due to windage, i.e., direct wind drag on the portion of the drifter above the water; to surface wave rectification, a spurious effect; to Stokes drift; to wind-driven Ekman currents; to Langmuir cells and to any other highly sheared, generally ageostrophic, horizontal currents that may occur near the surface. Although the water-following capabilities of the CARTHE, CODE and SVP drifters have already been studied [9,10,11], the new in situ measurements in a wider range of wind speed considered here provide new insights on the comparison between drifter-inferred currents and some practical guidance on how to use data of different drifters when operated in the same experiment at sea.

Simultaneous observations obtained from collocated pairs of different drifters are used to compute velocity differences and to relate them to surface wind and wave data from in situ measurements and climatological products. Additionally, velocity data from an Acoustic Doppler Current Profiler (ADCP) mounted on a CODE drifter are exploited to measure the velocity shear in the upper water column below the drifter. The main goal of this study is to quantify the differences between drifter velocity measurements as a function of wind speed and surface wave height.

A brief description of the CARTHE, CODE and SVP drifters is provided in Section 2, along with details about their deployments in the Alboran Sea (southwestern Mediterranean) and the ADCP measurements of relative currents. This section also includes information about ancillary in situ data of wind and waves from ship and drifters and on climatological model products of wind and waves. The methodology used to compare the currents measured by the drifters is also explained. The differences between the currents measured by the three drifter types and the measurements of relative currents below a CODE drifter are shown in Section 3, with particular focus on the downwind and down-wave components. Section 4 contains a discussion of the results, a comparison with previous findings. Main conclusions are provided in Section 5.

## 2. Data and Methods

### 2.1. CARTHE, CODE and SVP Drifters

The CARTHE drifter was developed to be a compact and quasi-biodegradable instrument [10]. It includes a top component in the form of a “donut” attached to a rigid drogue in a cross-shape extending about 60 cm below the sea surface. Tank experiments have demonstrated that the CARTHE drifter slip with respect to the mean Lagrangian currents in the first 60 cm below the surface amounts to less than 0.5% of the wind speed and does not exceed 3 cm/s in wind speed up to 23 m/s [10]. Furthermore, pairs of CODE and CARTHE drifters deployed together in the open ocean showed that the difference between the two drifter velocities is bounded by 0.25 cm/s. The drag area ratio is defined as *C_w_A_w_*/*C_a_A_a_*, where *C_a_* represents the drag coefficient of the elements of the drifter above the water surface (with cross-section area *A_a_*) and *C_w_* indicates the drag coefficient of the elements below the water surface (with cross-section area *A_w_*). For the CARTHE drifter, it is about 12 (Guigand, Personal communication).

The CODE drifter [12] measures the currents within the top meter of the water column. It is composed of a 1 m-long negatively buoyant tube with four drag-producing sails extending radially from the tube and four small spherical surface floats to provide buoyancy [13]. The tube is vertical, and the top of the sails is about 30 cm below the sea surface [14]. Poulain and Gerin [11] demonstrated that CODE drifters follow the currents with an accuracy of about 3 cm/s, even under strong wind conditions. The slippage produced by the wind and waves was estimated to be 0.1% of the local wind speed. Wind-driven currents measured by CODE drifters were found to be downwind (and also to the right of the wind vector) with about 1% of the wind speed [15]. The drag area ratio of the CODE drifter is about 30 [14].

The SVP drifter (see [16] for a recent description) is the standard drifter of the Global Drifter Program [17]. It consists of a spherical surface buoy (35 cm diameter) tethered to a weighted holey-sock drogue that allows it to track the horizontal currents in the water column between 12 and 18 m nominal depths. A strain gauge mounted on the drifter’s hull measures the tension of the tether connection to monitor the drogue presence. Direct measurements of the water-following capabilities of the SVP have shown that they follow the water near 15 m depth to within 1 cm/s in 10 m/s winds [9] when the drogue is attached. For the data used in this study, corresponding to periods less than a few months after deployment, we have checked that the drogue was always attached to the surface buoy. The drag area ratio of the SVP drifter is larger than 40 [9].

All drifters transmit their GPS data (and other ancillary data) via either Iridium (SVP) or GlobalStar (CODE and CARTHE) satellites, every 5 or 10 min, with typical accuracy of 10 m or better (e.g., [10]).

### 2.2. Other Drifters

Other drifters were used to provide ancillary in situ data on surface waves and relative currents in the upper water column.

The Directional Wave Spectrum Drifter (DWSD^TM^; [18,19]) is essentially an SVP for which the drogue is replaced by a small (~50 cm) stabilizing chain. It is equipped with a high-performance GPS engine paired with software algorithms for onboard computation of the directional wave spectrum of the surface waves. It transmits positions and surface wave statistics (significant wave height, peak and mean period, etc.) to the Iridium satellites every hour.

A prototype CODE drifter was fitted with a Nortek Aquadopp acoustic velocimeter and a Nortek Aquapro ADCP (respectively, at its top and bottom extremities) to measure the horizontal relative flow around and below the drifter, respectively [11,20]. Both instruments were programmed to transmit acoustic signals at 2 MHz and with an averaging interval of 1 s. The Aquapro ADCP at the bottom of the drifter (looking down) was set up with 20 vertical cells of 1 m. It includes ancillary sensors such as a tilt meter to measure its pitch and roll, a compass to record its orientation, a pressure sensor to measure the depth of the instrument and a thermistor. This drifter is referred to as the CODE ADCP hereafter.

### 2.3. Drifter Deployments

A large number of CARTHE, CODE, SVP and DWSD^TM^ drifters were deployed in the Alboran Sea (southwestern Mediterranean Sea) during two cruises of the Coherent Lagrangian Pathways from the Surface Ocean to Interior (CALYPSO) program sponsored by the U.S. Office of Naval Research [21]. The first cruise (CALYPSO 2018) was conducted in late spring 2018 [22] and involved 84 drifters. In early spring 2019, more than 180 drifters were deployed during the second cruise (CALYPSO 2019; [23]). Most drifters were deployed in tight clusters in order to measure the near-surface currents and related differential kinematic properties (e.g., vorticity and divergence) at scales as small as 1 km [8,24,25]. Several DWSD^TM^ drifters were deployed to measure surface wave characteristics in the study area. In particular, two units (14670 and 14680) were deployed near the CODE ADCP drifter during the CALYPSO 2019 experiment.

The CODE ADCP drifter was deployed several times during the CALYPSO 2019 experiment [23]. Unfortunately, the Aquadopp at the top of the drifter did not provide useful data. Only the data collected by the CODE ADCP on 9 April 2019 are considered in this study.

### 2.4. Drifter and ADCP Data Processing

The drifter position data were processed with standard methods for editing, kriging interpolation and low-pass filtering and interpolation [26]. Velocities were estimated by finite differencing of successive interpolated positions (central difference over 1 h). The position and velocity data are available at 0.5 h intervals. Low-pass filtering (hamming with cut-off period of 36 h) was applied to remove the high-frequency variability such as tidal and inertial currents. The filtered data were then sub-sampled every 6 h. Both un-filtered and filtered data sets are used in this study. Using a GPS position random error of 10 m and a time interval of 1 h, we estimated an error on the velocity of the order of 1 cm/s. The error on the low-pass filtered velocities is even less because the filtering removes most of the high-frequency GPS position errors.

The radial velocities measured by each ADCP beam were combined to estimate the currents in the upper layer of the sea below the drifter with an accuracy of about 1 cm/s. The tilt and compass data of the ADCP were then used to convert the data into the two components (zonal and meridional) of relative horizontal currents at distances from the drifter ranging in 1.4–20.4 m, with a resolution of 1 m. The tilt data were also used to convert these distances into depths below the sea surface, assuming that the pitch/roll motion is with respect to the drifter center of mass approximately at 0.8 m depth. The center of the deepest cell ranged between 13.6 and 21.7 m. The velocity profiles were finally interpolated at common depths between 3 m and the depth of the deepest cell (up to 20 m), with an interval of 1 m.

### 2.5. Estimation of Stokes Drift from DWSD^TM^ Data

The DWSD^TM^ returns the first five coefficients of the truncated Fourier series of the surface wave variance spectrum [18] and allows the estimation of the directional variance spectrum, which is defined as the product of a one dimensional wave spectrum, *E_var_*, and a directional spread function, *D_var_*:

(1) $$S_{var}\left(f,\theta \right)=E_{var}\left(f\right)\cdot D_{var}\left(f,\theta \right)$$

where *θ* is the direction of the wave vector at a given frequency *f* and *D_var_* is approximated by the order 2 truncated Fourier transform (e.g., [27]). Because of the low order of approximation, *D_var_* can assume negative values, and therefore, a weighted form, proposed by Longuet-Higgins [28], that ensures the power spectral density is always positive, is considered in this study, although it has the effect of diffusing the wave energy around the main peaks.

Following Kenyon [29], in absence of breaking waves and for deep water waves, the Stokes drift can be computed as:

(2) $$\vec{u\left(z\right)}=\frac{16\pi^{3}}{g}\iint f^{3}\;\left(cos\left(\theta \right),sin\left(\theta \right)\right)\;S_{var}\left(f,\theta \right)\;e^{\frac{8\pi^{2}f^{2}}{g}z}d\theta df$$

where *g* is the gravity and *z* is the water depth, negative downward.

Equation (2) prescribes that the Stokes drift decays exponentially with increasing depth and is larger for short wavelengths.

### 2.6. Ancillary Data and Climatological Products

During the CALYPSO 2019 experiment, data of wind speed and direction were collected with a Gill Windsonic anemometer (part of the BATOS meteorological system of Meteo France) on board the Research Vessel (RV) Pourquoi Pas?. The GPS ship location and the wind data are available with a sampling rate of 1 Hz.

The European Centre for Medium-Range Weather Forecasts (ECMWF) fifth generation atmospheric reanalysis of the global climate (ERA5) provides hourly estimates of a large number of atmospheric, land and oceanic climate variables. ERA5 products were obtained in the southwestern Mediterranean for the time periods of the CALYPSO 2018 and 2019 experiments. Winds at 10 m height (zonal and meridional components) and surface waves’ characteristics (significant wave height and mean direction) are available on 0.25° and 0.5° grids, respectively. As it will be shown later, the ERA5 wind and wave products are underestimated when compared to direct in situ measurements from ships in the southwestern Mediterranean Sea. Wind speed is typically underestimated by about 5 m/s, and significant wave height by 0.5 m. In addition, ERA5 wind speeds and significant wave heights appeared well correlated, meaning that the surface waves were mainly generated by the local winds.

### 2.7. Comparison Methodology

**Figure 1:**
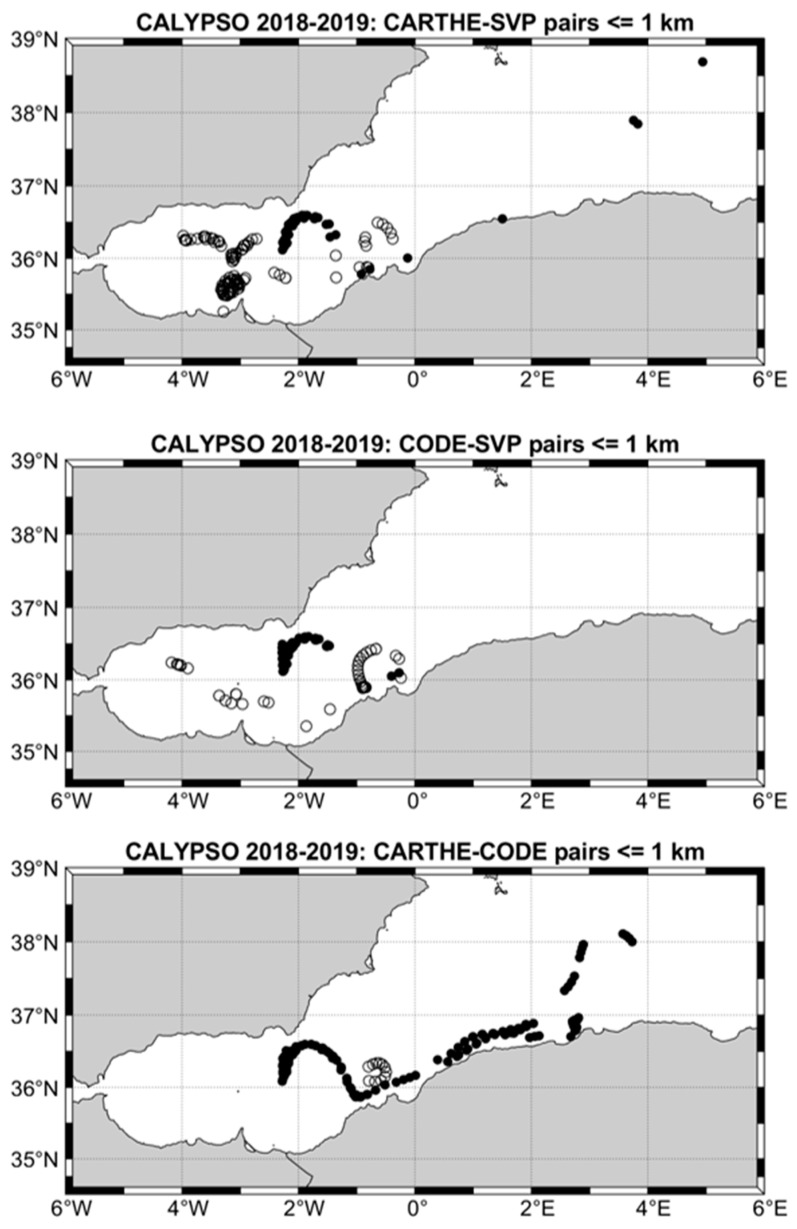
Locations of the CARTHE-SVP, CODE-SVP and CARTHE-CODE drifter pairs with separation distance less than 1 km: CALYPSO 2018 (black dots) and CALYPSO 2019 (circles).

First, the low-pass filtered drifter data (positions and velocities) were considered along with the ERA5 wind and wave data filtered in the same way. Nearly collocated and simultaneous pairs of CARTHE-SVP, CODE-SVP and CARTHE-CODE data were searched in the drifter dataset. The maximum distance between the two drifters in the pair was set to 1 km (see Appendix A for a discussion on this threshold and on the influence of horizontal shear). The locations of these pairs in the southwestern Mediterranean Sea are shown in Figure 1. Using 1 km, the number of pairs ranges between 122 and 262 (see Table 1 and Table 2 and Figure 1). The low-pass filtered ERA5 wind and wave data were interpolated at the pair positions. The drifter velocity difference was then projected in the direction of, and perpendicular to, the wind or waves.

Second, for the period corresponding to the successful deployment of the CODE ADCP, on 9 April 2019 between 8:45 and 20:47 UTC, the non-filtered drifter data at 0.5 h intervals were considered. Pairs of CARTHE-CODE ADCP and CARTHE-SVP drifters near the CODE ADCP and with distance less or equal to 1 km were found. The wind data collected on the research vessel were used along with the drifter velocity differences. The distance between the research vessel and the drifters was less than 25 km. The wave data of two DWSD^TM^ drifters in the vicinity (<17 km) of the CODE ADCP were also used. As before, the drifter velocity differences were projected in the downwind and cross-wind directions.

## 3. Results

### 3.1. Comparison Using Low-Pass Filtered Data

**Figure 2:**
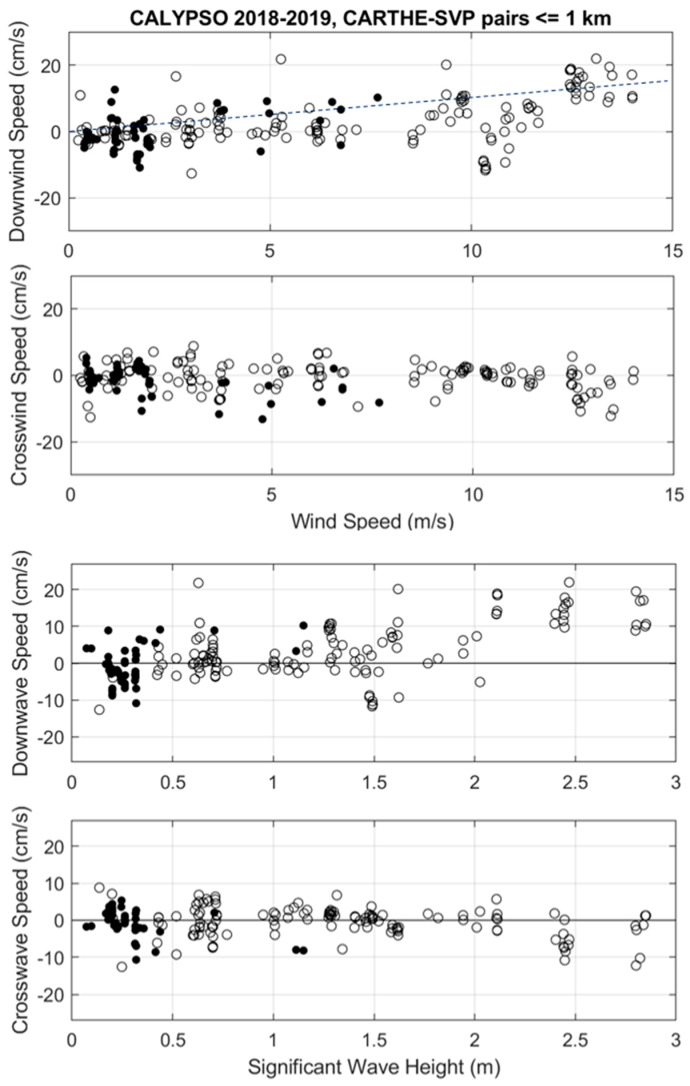
Down- and cross-wind components of velocity difference between CARTHE and SVP drifters versus ERA5 wind speed (**top panels**) and significant wave height (**bottom panels**): CALYPSO 2018 (black dots) and CALYPSO 2019 (circles). The 1% slope is shown as a dashed line in the top panel for reference.

For the comparison between CARTHE and SVP, the range of wind speed and significant wave height are 0–14 m/s and 0.0–2.9 m, respectively (Figure 2). The downwind velocity difference mostly increases with wind speed for wind speed larger than 8 m/s during CALYPSO 2019. The maximum downwind velocity difference is ~22 cm/s at wind speed of 13 m/s and significant wave height of ~2.5 m (Figure 2). Considering all wind speeds, the scatter is rather large with a standard deviation (STD) of ~7 cm/s and a mean of 1.8 cm/s (Table 2). In the direction perpendicular to the wind, the scatter is slightly reduced (STD = 4 cm/s), and the maximum difference is ~13 cm/s. The mean of −0.6 cm/s indicates some tendency of the CARTHE-SVP velocity difference to go to the left of the wind and waves (looking downwind). Note that in both directions, the scatter of velocity differences can be substantial at low wind speed (<5 m/s), especially for CALYPSO 2018.

**Figure 3:**
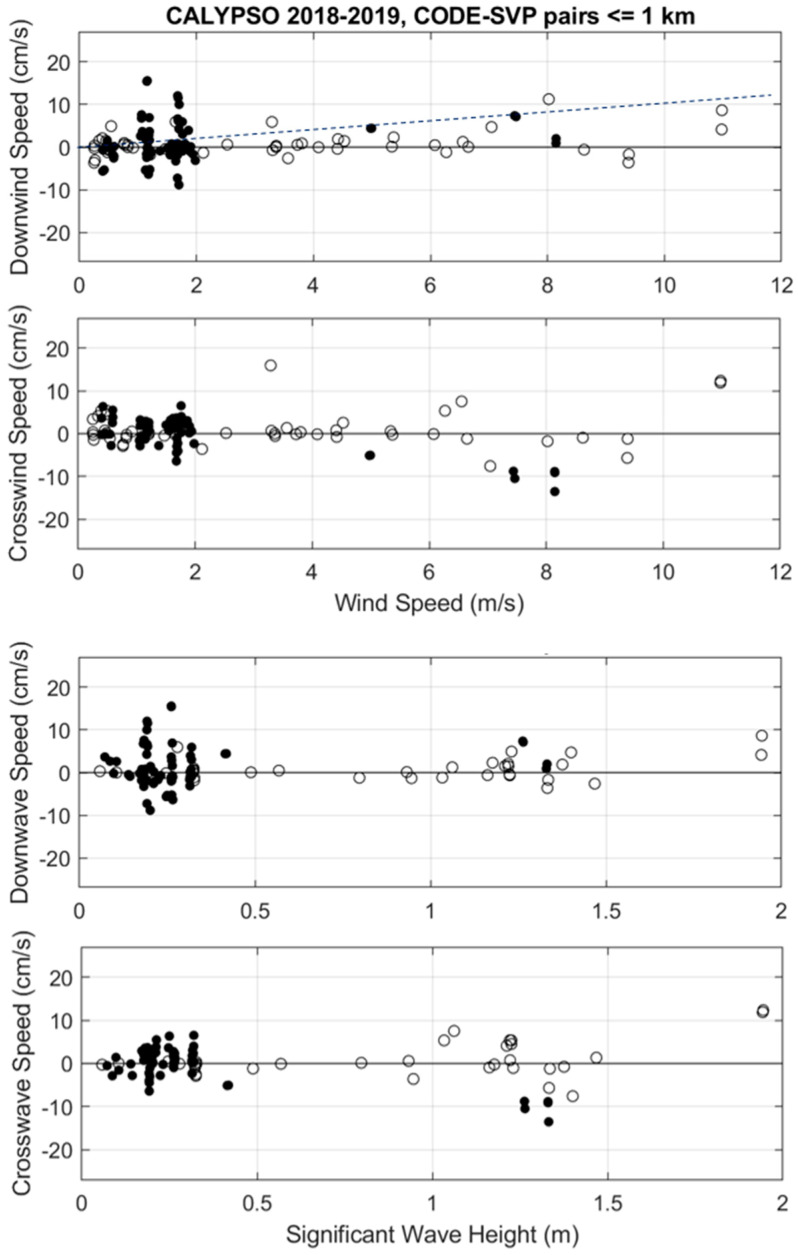
Down- and cross-wind components of velocity difference between CODE and SVP drifters versus ERA5 wind speed (**top panels**) and significant wave height (**bottom panels**): CALYPSO 2018 (black dots) and CALYPSO 2019 (circles). The 1% slope is shown as a dashed line in the top panel for reference.

For the CODE drifters compared to the SVP, the scatter at low winds in 2018 is even more important, with a maximum velocity difference of ~15 cm/s (Figure 3). However, the 2019 data reveal a general positive trend with increasing wind speed up to ~11 m/s with a maximum of ~10 cm/s. This relationship is also evident as a function of surface wave height (Figure 3). In the cross-wind direction the scatter is as large as for the CARTHE (STD = 4 cm/s) without significant mean or trend (Table 2).

**Figure 4:**
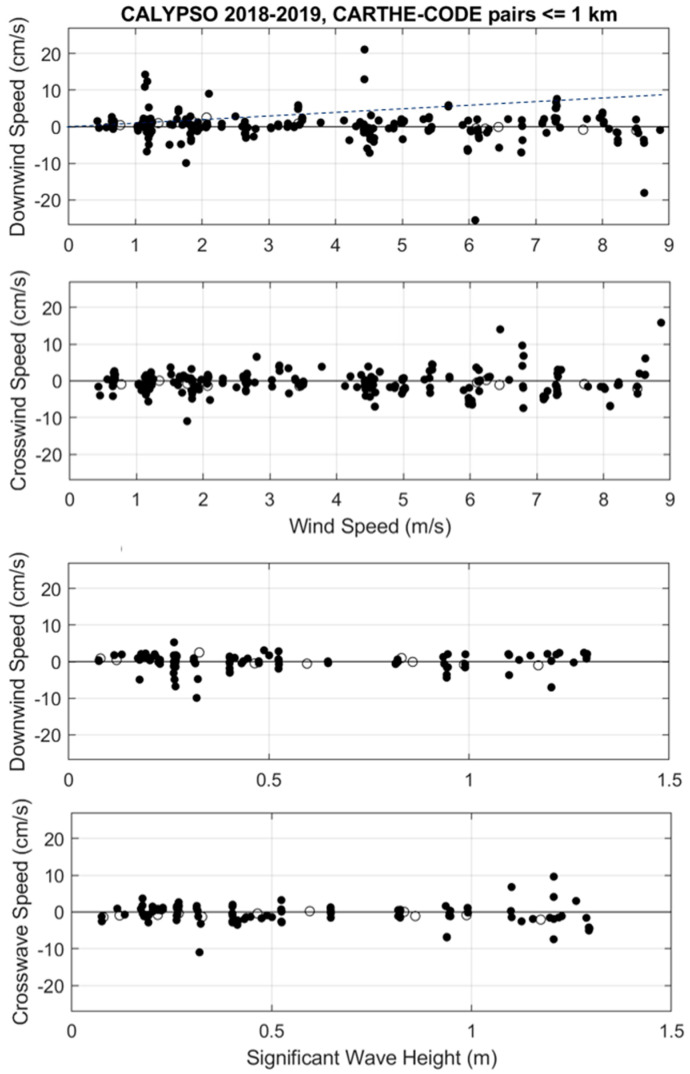
Down- and cross-wind components of velocity difference between CARTHE and CODE drifters versus ERA5 wind speed (**top panels**) and significant wave height (**bottom panels**): CALYPSO 2018 (black dots) and CALYPSO 2019 (circles). The 1% slope is shown as a dashed line in the top panel for reference.

Regarding the direct comparison between CARTHE and CODE drifters, there is no evidence of dependence on wind speed or surface wave height for the meteo-marine conditions encountered during the CALYPSO experiments: winds less than 9 m/s and significant wave height less than 1.3 m (Figure 4). The mean velocity difference in both down- and cross-wind directions is essentially zero, with an STD of 3–4 cm/s (Table 2).

### 3.2. Comparison Using Non-Filtered Data on 9 April 2019

**Figure 5:**
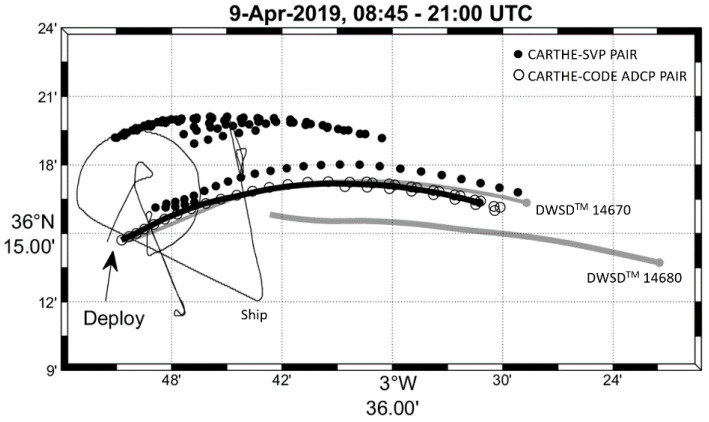
Trajectories of the CODE ADCP drifter (thick black) and positions of the CARTHE-SVP (black dots) and CARTHE-CODE ADCP (circles) pairs on 9 April 2019 between 8:45 and 20:47 UTC. Trajectories of the DWSD^TM^ drifters (14670—thin gray, and 14680—thick gray) drifters and RV Pourquoi Pas? track (thin black) for the same period. All drifters are moving eastward.

On 9 April 2019, a CODE drifter equipped with an ADCP provided data of the relative currents below the drifters to about 20 m depth. Thus, it is interesting to compare the drifter velocities around this CODE ADCP during its deployment period (about 12 h) using the drifter and ship wind data interpolated every 0.5 h. The trajectories of the CODE ADCP, DWSD^TM^ drifters and RV Pourquoi Pas? track between 08:45 and 20:47 UTC on 9 April 2019 are shown in Figure 5. The positions of the CARTHE-SVP and CARTHE-CODE ADCP pairs with separation distance less or equal to 1 km are also shown. During that period, all drifters were moving eastward in the northern limb of the Western Alboran Gyre with speeds as large as 1 m/s.

**Figure 6:**
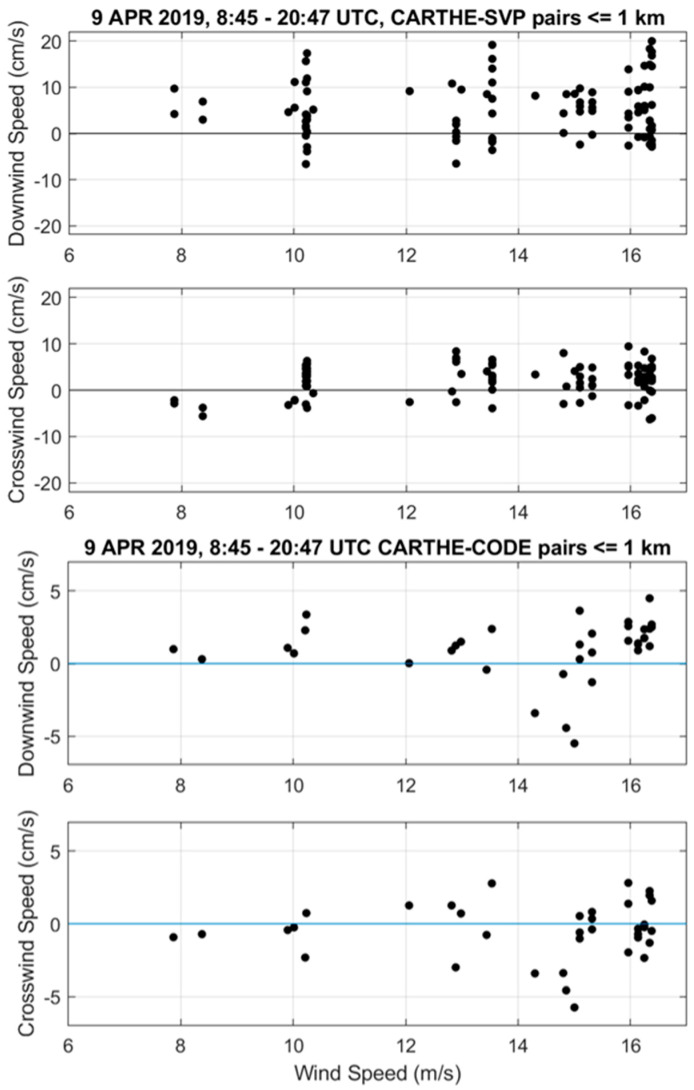
Down- and cross-wind components of velocity difference between CARTHE and SVP (**top panels**) and CARTHE and CODE ADCP (**bottom panels**) drifters versus wind speed on 9 April 2019 between 08:45 and 20:47 UTC.

The comparison between the drifter velocities, projected in the down- and cross-wind directions, show results similar to those obtained with the low-pass filtered data. Indeed, for the CARTHE-CODE pairs, the velocity differences remain bounded by 5 cm/s, and the downwind component has a slight tendency to increase with wind speed (Figure 6). For the CARTHE-SVP pairs, the downwind velocity difference increases to ~20 cm/s for winds near 17 m/s (Figure 6). If we consider all the wind speeds (Table 3), there is a significant mean offset downwind (~5 cm/s) and to the right of the wind (looking downwind; ~2 cm/s). The scatter is generally higher (STD up to 6 cm/s, see Table 3), presumably due to the inclusion of high-frequency signals such as inertial and tidal currents.

**Figure 7:**
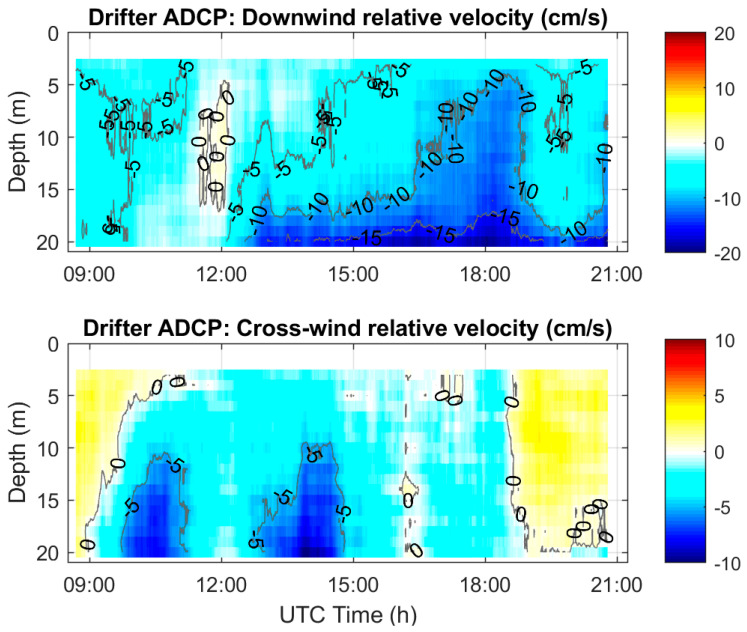
Color-coded contour diagrams of the relative currents measured by the CODE ADCP on 9 April 2019, projected in the down- (**top**) and cross- (**bottom**) wind directions. Positive is down-wind and to the right of the wind (looking downwind).

The relative currents measured by the CODE ADCP were interpolated every meter and were averaged using a 1 h running window and then projected in the down- and cross-wind directions (Figure 7). In the downwind direction (which is practically eastward), relative currents are weak and vertically homogeneous around 12:00 UTC, followed by the increase of the vertical shear to 10–15 cm/s for the velocity difference between 3 and 20 m. The relative downwind velocity magnitude at 20 m approached 20 cm/s after 18:00 UTC. In the cross-wind direction, relative velocities are bounded by −10 and 2 cm/s. They are mostly negative (to the left of the wind) before 19:00 UTC and positive after. Some shear is evident before 15:00 UTC, but after that time, the cross-wind relative currents are rather homogeneous over the entire water column above 20 m depth.

**Figure 8:**
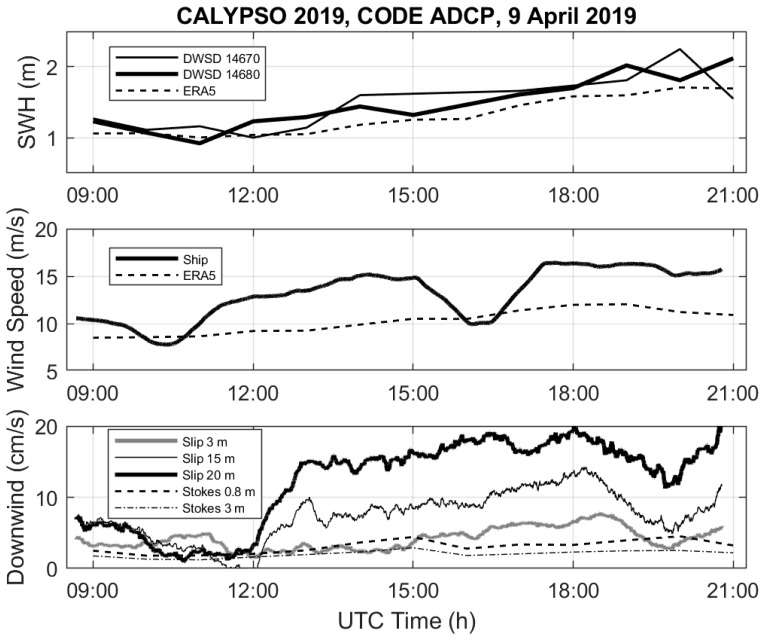
Significant wave height **(top panel**; DWSD^TM^ 14670—thin, DWSD^TM^ 14680—thick, ERA5—dashed), wind speed (**middle panel**; ship—solid, ERA5—dashed) and downwind slip of the CODE with respect to the currents at 3 m (gray), 15 m (thin black) and 20 m (thick black), measured by the ADCP versus time on 9 April 2019 (**bottom panel**). The downwind slips ue to the Stokes drift shear between 0.8 m and 3 m (dashed–dotted) and 15 m (dashed) are also shown.

The downwind drift (or slip) velocity of the CODE ADCP with respect to the velocities at 3, 15 and 20 m depths, which are the mean depths of the first and last cells and the nominal depth of the SVP drogue, are depicted versus time in Figure 8, along with the hourly wind and wave conditions. Both in situ direct measurements and ERA5 products of wind speed and significant wave height, although affected by an offset, reveal an increase of wind speed from 10 to 15 cm/s, and from 1 to 2 m in significant wave height, during the CODE ADCP deployment period. A substantial shear in the horizontal currents dominates between 13:00 and 19:00 UTC, with relative currents at 20 m exceeding 10 cm/s, while the speed at 3 m remains bounded by 8 cm/s. The CODE ADCP downwind drift with respect to the currents at 15 m (20 m) depth reached a maximum of ~15 cm/s (~20 cm/s) after 18:00 UTC.

The Stokes drift was estimated using Equation (2) with the directional spectral data from the DWSD^TM^ drifter 14670. On 9 April 2019 between 09:00 and 21:00 UTC, the maximum Stokes drift magnitude at the surface is 7 cm/s (not shown). Below 10 m depth, it is always negligible. The relative downwind (down-wave) current due to Stokes drift at 3 and 15 m with respect to the value at 0.8 m (depth of the CODE center) is shown in Figure 8. Maximum values are 3 and 5 cm/s for 3 and 15 m, respectively.

The relative ADCP currents and their vertical shear shown in Figure 7 and Figure 8 do not appear to be well correlated with the local winds and wave height. In particular, the weak vertical shear around 12:00 UTC in both down- and cross-wind directions does not correspond to any specific signal in the wind speed and wave height. Other CALYPSO 2019 measurements by an ADCP mounted on a Wirewalker and by the ship-mounted ADCP of the RV Pourquoi Pas? on 9 April 2019 [30] confirm this temporal evolution of the vertical shear of horizontal currents that is not directly related to the local winds.

**Figure 9:**
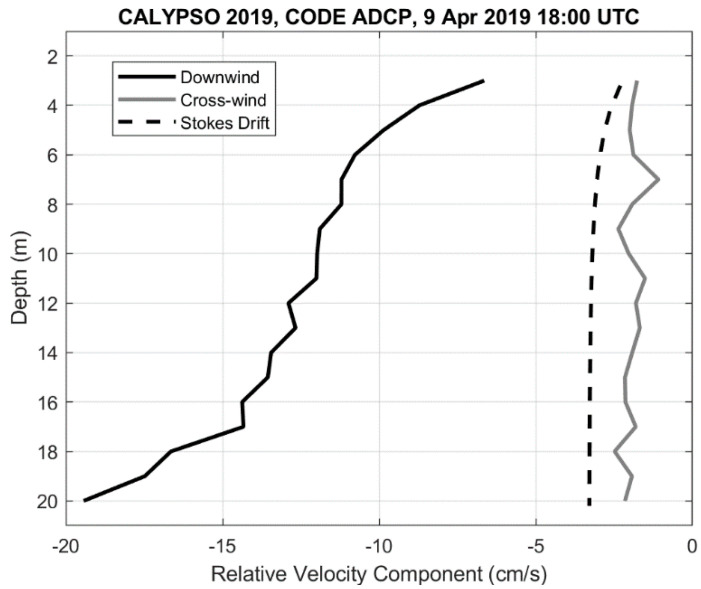
Hourly average of down-wind (black) and cross-wind (gray) components of the relative velocity measured by the CODE ADCP versus depth, centered at 18:00 UTC on 9 April 2019. The Stokes drift estimated relative to the value at 0.8 m depth is shown with a dashed curve.

If we focus on the hour centered at 18:00 UTC, the downwind relative current varies between ~−7 cm/s about 3 m below the surface to ~−19 cm/s at 20 m depth (Figure 9). Most of the shear is above 6 m and below 17 m depth (about 1–2 cm/s over 1 m). These results can be interpreted as velocity differences, i.e., the CODE ADCP moves downwind by about 7 cm/s with respect to the current at 3 m and by about 14 cm/s with respect to the currents at 15 m. The relative current due to Stokes drift is also shown in Figure 9. It varies between −2.3 cm/s at 3 m and −3.3 cm/s at 20 m. In the cross-wind direction, the relative currents are rather constant with depth, with a value between −2 and −3 cm/s. These results show that the CODE ADCP moves to the right of the wind (looking downwind) compared to the currents in the upper water column.

## 4. Discussion

The currents measured by CARTHE, CODE and SVP drifters in the southwestern Mediterranean during the CALYPSO 2018 and 2019 experiments were compared. Even though these experiments were not designed to compare the drifters, the measurements obtained in a wide range of wind speed [0–17 m/s] allowed us to further assess the water-following characteristics of the instruments. Surface waves, mainly generated by the local wind, were essentially propagating in the downwind direction, and their amplitude reaching a maximum of 3 m significant wave height, increased with the wind speed. Differences in drifter velocity in the down- and cross-wind directions were examined as a function of wind speed and significant wave height measured in situ or provided by the ERA5 climatological model. Scatter diagrams (e.g., Figure 2 and Figure 6) reveal positive trends in the downwind direction when CARTHE and CODE drifters are compared to SVP drifters. Maximum differences of more than 20 cm/s are found for a wide range of wind speed between 5 and 15 m/s. However, the increase versus wind speed is mostly evident for winds stronger than 8 m/s. For these strong winds, the CARTHE-SVP and CODE-SVP velocity differences are 0.5–1% of the ERA5 wind speed, which is similar or somewhat less than the slope of 1% found by Poulain et al. [15,31] and Pazan and Niiler [32] for the un-drogued SVP with respect to the drogued SVP drifter. This is expected, since the surface buoy of the SVP has more direct wind drag above the sea surface compared to the CARTHE and CODE drifters. Thus, we can conclude that the typical difference between the surface drifters and the drifters drogued at 15 m is 0.5–1% of the wind speed, i.e., a difference of about 5–10 cm/s in 10 m/s winds. This is less than the classical “slip law” of 3% [33,34,35], which is sometimes used operationally to estimate the drift of oil slicks or floating objects/bodies in addition to numerically modelled or climatological near surface currents.

However, there is large scatter around this rule and individual relative speeds can even be as large as 20 cm/s in wind speeds near 5 m/s (Figure 2). The large scatter at low wind speed (<10 m/s) is most likely due to the horizontal shear between the drifters separated by less than 1 km (see Appendix A for a discussion). ADCP measurements also show relative currents at 15 m depth below the CODE drifter (see Figure 7 and Figure 8) that can be as large as ~15 cm/s in winds near 15 m/s.

In the direction perpendicular to the wind, the interpretation of the results is difficult, but differences between CARTHE/CODE and SVP rarely exceed 10 cm/s, and their means are generally not significantly different from zero (see Figure 2 and Table 2). The non-filtered data on 9 April 2019 revealed that the CARTHE has a tendency to move to the right of the wind, compared to the SVP (Figure 6 and Table 3). However, the ADCP measurements below the CODE indicated mostly negative cross-wind relative currents (Figure 7).

The currents measured by CARTHE and CODE drifters were also compared with each other (Figure 4 and Figure 6, Table 2 and Table 3). The results obtained for the wind/wave conditions of the southwestern Mediterranean during the CALYPSO 2018 and 2019 experiments do not show significant differences. Only some tendency of the CARTHE to move more downwind is sometimes seen (see, for instance, in Figure 6). This might be expected, since the CARTHE drifter is closer to the sea surface and has a smaller drag area ratio compared to the CODE. We therefore conclude that the two types of surface drifters, CARTHE and CODE, measure the currents in approximately the same way and that their data can be combined to investigate the surface kinematics and dynamics [24,25,36].

The individual contributions to the velocity difference between CARTHE/CODE and SVP drifters include the effect of direct wind drag and wave rectification and the shear in the upper layer related to Stokes drift, Ekman spiral, Langmuir cells and other ageostrophic currents. We have shown that, for the relatively large wave conditions measured in the southwestern Mediterranean Sea (up to 3 m significant wave height), the exponentially decaying Stokes drift contribution to the total surface current can only partially explain this difference. The shear between 0.8 and 3 m that reaches 7 cm/s includes a contribution of 3 cm/s due to Stokes drift (Figure 8 and Figure 9). Between 0.8 m and 15 m, the Stokes drift can produce a velocity difference of about 5 cm/s compared to 14 cm/s observed, that is, about 30–40% of the measured velocity difference.

Additional shear can be due to the wind-induced Ekman flow. From the Ekman theory, a wind speed of 17 m/s and a fully developed Ekman layer with a depth of 40 m should induce a velocity difference between 0.8 and 3 m of ~0.9 cm/s. For a depth of 20 m, it should be ~3.3 cm/s. Since these currents are rotated at an angle in excess of 45° to the right of the wind in the northern hemisphere, they correspond to a maximum downwind component of the Ekman current difference between 0.8 m and 3 m of ~0.5 cm/s and 2.2 cm/s for Ekman depths of 20 and 40 m, respectively. Therefore, the sum of the Ekman Current and Stokes drift may still not be sufficient to explain the relative currents measure with the ADCP mounted on the CODE drifter. We conclude that spurious wave rectification effects on shallow drogued drifters (e.g., CARTHE and CODE drifters) may also play a role in explaining the discrepancy and that in the presence of large waves, the use of such drifters may introduce a source of errors of a few cm/s for the measured down-wave currents.

## 5. Conclusions

To conclude, we have demonstrated that:

(1) Despite their different designs (e.g., depth and drag-area-ratio differences), the CODE and CARTHE drifters measure the currents in the first meter below the sea surface in approximately the same way, and they can be combined to calculate ocean surface velocity statistics and dynamics.
(2) The surface drift of CODE and CARTHE drifters is typically downwind with 0.5–1% of the wind speed, when compared to the SVP at 15 m depth. The veering of the CODE/CARTHE currents to the right of the wind was only partially confirmed by the observations. The large scatter in velocity differences between CODE/CARTHE and SVP for all wind and sea state conditions is principally due to vertical and horizontal shears not related to the wind.
(3) Ekman theory and wave-induced Stokes drift dynamics only partially explain the observed vertical shear of horizontal currents measured by the CODE ADCP in the upper sea.

## Figures and Tables

**Table 1:** Numbers of pairs of drifters with distance less or equal to 1 km using the CALYPSO 2018 and CALYPSO 2019 low-pass filtered datasets.

Experiment	Drifters	Pairs	Dates
CALYPSO 2018	CARTHE-SVP	62	1 June–8 July 2018
	CODE-SVP	72	1–5 July 2018
	CARTHE-CODE	250	1–17 June 2018
CALYPSO 2019	CARTHE-SVP	154	5–18 April 2019
	CODE-SVP	50	31 March–18 April 2019
	CARTHE-CODE	12	14–17 April 2019

**Table 2:** Statistics of the difference between pairs of drifters using low-pass filtered drifter and wind data.

Experiment	Pairs	Downwind (cm/s)			Cross-Wind (cm/s)		
		Mean	STD	Min/Max	Mean	STD	Min/Max
CARTHE-SVP	216	1.8	7.3	−12.6/21.9	−0.6	4.0	−13.2/8.8
CODE-SVP	122	1.0	4.1	−8.8/15.5	0.4	4.0	−13.5/15.9
CARTHE-CODE	262	0.2	3.9	−25.5/21.1	−0.5	2.9	−11.0/15.8

**Table 3:** Statistics of the difference between pairs of drifters on 9 April 2019 between 08:45 and 20:47 UTC.

Experiment	Pairs	Downwind (cm/s)			Cross-Wind (cm/s)		
		Mean	STD	Min/Max	Mean	STD	Min/Max
CARTHE-CODE	36	1.0	2.1	−5.5/4.5	−0.5	2.0	−5.7/2.8
CARTHE-SVP	97	5.3	6.2	−6.6/20.0	2.2	3.5	−6.3/9.4

**Figure A1:**
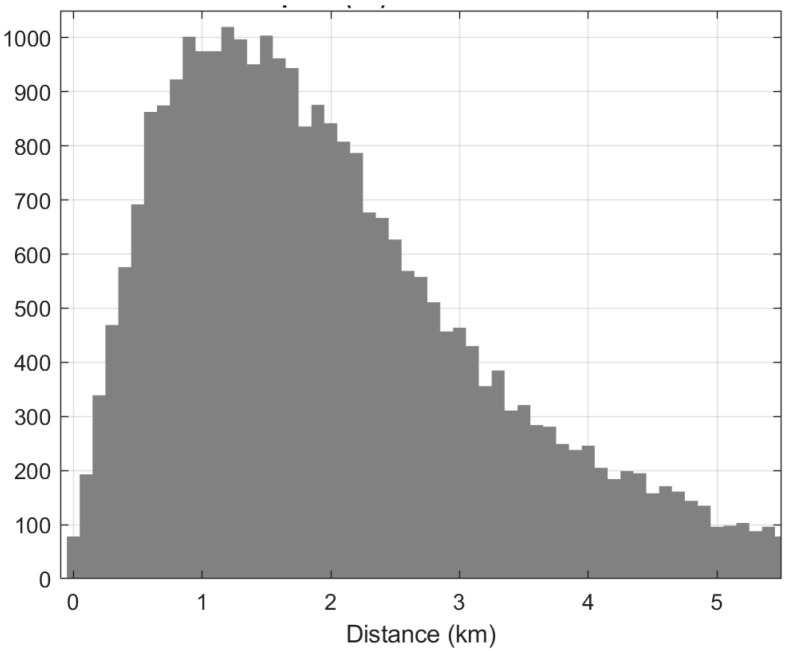
Histogram of the distance between low-pass filtered (hamming 36 h, sampled every 6 h) and interpolated (every 0.5 h) drifter positions, using the entire CALYPSO drifter dataset in the southwestern Mediterranean Sea.

**Figure A2:**
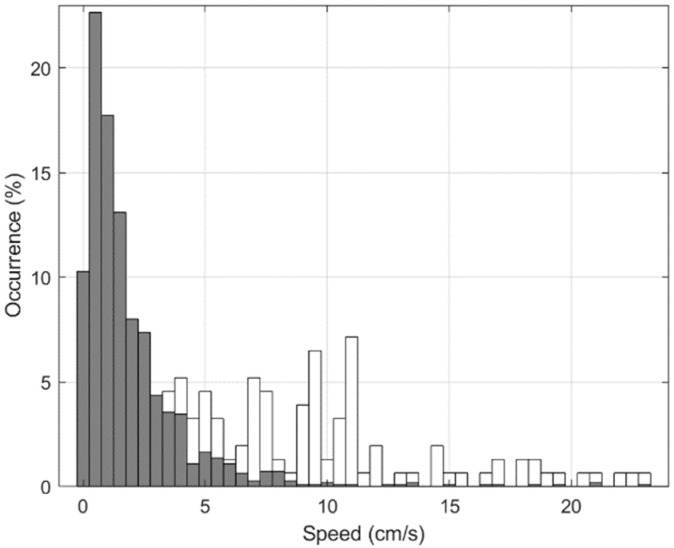
Normalized histograms of the magnitude of the velocity difference using low-pass filtered drifter data and a separation distance ≤ 1 km: 1099 CARTHE–CARTHE pairs in CALYPSO 2018–2019 (gray bars) and 154 CARTHE-SVP pairs in CALYPSO 2019 (white bars).

## Data Availability

The drifter data used in this study are available on the CALYPSO web cloud. The ECMWF ERA5 products can be downloaded from the Copernicus Climate Data Store (cds.climate.copernicus.eu (accessed on 11 November 2021)).

## Appendix A. Distance between Drifters and Horizontal Shear

Before discussing the effects of the maximum separation distance used to search for drifter pairs on the statistics presented in this paper, it is worthwhile to estimate the typical distance between raw and low-pass filtered (hamming with 36 h cutoff) drifter positions. Assuming that the distance between the raw positions sampled every 5 or 10 min and the interpolated positions every 30 min is small and comparable to the GPS accuracy (~10 m), we have computed the distance every 6 h between the interpolated and low-pass filtered position time series for all the CALYPSO drifters in the southwestern Mediterranean Sea (285 drifters). The distribution of such a distance is show in Figure A1. The mean is 2.2 km, and the mode is 0.9 km, whereas the maximum distance is ~19 km. The accuracy of the filtered drifter positions is, therefore, a few kilometers.

If low pass-filtered positions are used to find pairs, a threshold maximum distance less than 1 km does not make sense. Note that if we use the non-filtered positions in conjunction with the filtered velocities (to be more accurate in position), the statistical results presented in this paper remain essentially the same.

It is obvious that if we increase the threshold distance above 1 km, the numbers of pairs will increase and, in theory, the statistical results will be more robust. However, increasing the distance the velocity difference between drifters will include a significant increasing contribution from the horizontal shear of currents. As demonstrated in [24,33], this shear can be quite substantial at scale of a few kilometers in the southwestern Mediterranean. In order to assess the effect of the horizontal shear, we have calculated velocity difference statistics using pairs of identical drifter types. For instance, 1099 CARTHE–CARTHE pairs with distance less or equal to 1 km were found in CALYPSO 2018–2019 dataset. The normalized histogram of the magnitude of velocity differences is shown in Figure A2. Values are essentially bounded by 5 cm/s. For comparison, a similar histogram is also depicted for the 154 CARTHE-SVP of CALYPSO 2019 (corresponding to the circle symbols in Figure 2). As expected, the distribution is wider and includes values reaching 20 cm/s. Figure A2 shows that the horizontal shear over 1 km can produce velocity differences of up to 5–10 cm/s. Higher values (10–15 cm/s) are related to the local winds and waves.

In summary, given the above considerations, we believe that using a maximum distance less than or equal to 1 km between the drifters in the pairs is a good practical tradeoff, providing enough observations and limiting the effects of the horizontal shear on the results.
